# Proceedings of the Louisville Medical Club

**Published:** 1855-12

**Authors:** John Bartlett

**Affiliations:** Secretary


### ART. II.—PROCEEDINGS OF THE LOUISVILLE MEDICAL CLUB.

REPORTED BY JOHN BARTLETT, M. D., SECRETARY.

THE Louisville Medical Club met at the house of Dr. Bullitt on the evening of the 6th of August, 1855.

President Ewing in the chair, and the following members present: Drs. Bullitt, Bemiss, Goldsmith, Gross, Rogers, Richardson, Wible, Knight, Thomson, and Bartlett.

Dr. Bemiss' paper on croup was read as follows:

Instead of attempting to write a formal essay upon this subject, I shall read to the society the notes of a case of croup which I treated some months ago, and then offer some remarks upon the symptoms and treatment of croup, taking the case referred to as the type in my analysis. If, then, I assume this case to have been one of true membranous croup, as I think it undoubtedly was, all those varieties of the disease in which there is not evidence of false membrane, will be excluded from direct observation. I will notice these forms of the disease after I have detailed the case and come to speak of the diagnosis and probable pathology of membranous croup.

I was called at noon on the 21st of September, 1854, to see a child of Prenett, a Frenchman, living on Eleventh street, in an upper room, airy and comfortable. The father was a healthy, robust man; the mother a slender, delicate woman. The child was two years and three months old, well developed, though somewhat emaciated from diarrhœa during the previous summer, but for some weeks before this attack his bowels had been entirely regular. For several days past he had had symptoms of ordinary catarrh, with occasional difficulty in respiration. At the time of my visit he had some febrile symptoms, his pulse was between 120 and 130, though his skin was soft and not of unusual temperature; his throat externally seemed somewhat tumefied, internally the tonsils were evidently slightly enlarged, his tongue was furred and tip red; upon the surface of the fauces a whitish pellicular deposit was distinctly seen; he had the crowing respiration observable in membranous croup, and its dry, tight, embarrassed cough; his voice was sharp and whispering. For the sake of brevity I will give only such an abstract of the remaining notes as to show all the remedies employed and their manner of exhibition. On my first visit a sponge, tied to a piece of whalebone, was wet in a solution of nitrate of silver, 15 grains to the ounce, and applied to the fauces as freely as the struggles of the child would permit. This was repeated on the evening of the same day, and again the next morning, and then abandoned.

Calomel, combined with gum arabic, was given in grain doses every two hours till the bowels were well evacuated. After this, powders containing each a half grain of calomel, and a half grain of ipecac, and one grain of extract of hyoscyamus, were given at from four to six hours interval. Whenever active catharsis was threatened, the calomel was omitted and the hyoscyamus and ipecac continued alone.

As often as respiration became impeded from accumulation of tenacious mucus about the fauces, 5 grains of sulphate zinc were given, dissolved in a small quantity of water. This produced instant emesis, without any previous nausea or subsequent exhaustion. These emetics were repeated from one to four times in the twenty-four hours. On my first visit a warm poultice was ordered to the patient's throat, but it was found difficult to retain it during his restlessness, and when I saw him again a small napkin was folded, dipped in cold water and laid around the neck, a strip of oiled silk was placed over this, and a light silk handkerchief over them all. So soon as the napkin became dry, it was again wet in cold water and reapplied. This course, so far as relates to the water, was unremittingly pursued till the symptoms were decidedly mitigated. It was then abandoned and the throat occasionally rubbed with a liniment of turpentine, camphor, and olive oil. The parents were enjoined to suffer the boy to sleep whenever there was disposition to do so, and the symptoms were not urgent. They were instructed to nourish him well, and soft, boiled eggs, of which he was very fond, were freely allowed him. After the first few days, when the powers of life seemed disposed to flag, wine, whey, and weak toddy were given several times a day.

On the 27th of September, the sixth day of treatment, there was manifest relaxation in the severity of the symptoms—the respiration became less stridulous, and the cough more rattling. The patient expectorated several irregular fragments of concrete lymph, accompanied with muco purulent looking sputa. The hyoscyamus and calomel were now omitted, and two grains of Dover's powder, given during restlessness at night, also administered. From this period till the termination of the case a solution of carbonate of soda, with syrup of tolu, was also administered, from three to four times a day.

On the 2d of October, or eleventh day of treatment, the patient was discharged. At that time there was still copious expectoration; the voice was still but a feeble whisper, though the pulse was normal; the appetite was good, and disposition bright and playful. About two months afterwards his father brought him to my office. His health was then good, and his voice restored.

The symptoms diagnostic of membranous croup were, I think, strongly marked in this case. The respiration was laborious and stridulous; there was sinking of the soft parts of the thorax at each inspiration; the child's head was thrown back; his cough was sharp, barking, and ineffectual. It had that peculiar 'croupy' sound which we all so readily recognize, but are so little able to describe. His voice was a sharp whisper. One other notable symptom was the deposit upon the fauces. Dr. Ware found that in seventy-five cases of membranous croup where this was sought for, it failed to be present in only one. If this should correspond with the observations of the profession generally, it will be regarded as the most important point in the diagnosis of croup.

The only conditions of disease with which the above case could have been confounded, are those forms of croup in which the impediment to respiration arises from other causes than the deposit of false membrane in the trachea. These causes may be, first—Spasm of the glottis. Spasmodic croup may generally be recognized by the suddenness of its incursion. It may give rise to great alarm by the violence of its symptoms, but readily subsides when the irritating cause is removed. We may further distinguish it by the fact, that in membranous croup both expiration and inspiration are difficult; but in spasmodic croup the inspiration is attended with great effort, while the expiration is comparatively easy. The cough of spasmodic croup is louder, more hoarse and rancous, and the general muscular movements of the child are often convulsive. Still, it is the testimony of most observers that more or less spasm is connected with every case of membranous croup. We cannot otherwise account for the sudden exacerbations in that form of disease except by referring them to spasmodic action of the glottis. But when the spasm of membranous croup subsides, although there is some mitigation in the violence of the symptoms, still the cough and respiration are identical in character though less intense than they were during the exacerbation. On the contrary, when the spasm of spasmodic croup subsides, the child is comparatively or entirely relieved. 2. Croupy symptoms may be produced by profuse secretion of mucus in the air-passages, constituting what is often termed catarrhal croup. In this form of disease the respiration is never so dry and ringing as in membranous croup; the cough is looser and more rattling; the mucous râle generally pervades one or both lungs. There is, moreover, not that urgency of symptoms, nor the "concentration, as it were, of the child's whole mind to the one act of inspiration," that we observe in membranous croup. 3. Inflammatory croup, or that form of disease in which inflammation and tumefaction of the mucous membrane so narrow the rima glottidis as to interfere with respiration. The diagnosis between this affection and membranous croup is less strongly marked. There may be very close resemblance between the sounds of the cough and respiration in the two diseases, and the only positive point of diagnosis would be the appearance of the deposit upon the fauces in the one case, and its absence in the other. 4. A species of diptheritic affection of the throat sometimes follows measles and scarlatina, which may simulate membranous croup. In truth, this form of disease is attended with lymphatic deposits, but generally in small patches, and often covering spots of ulceration of the mucous membrane. The history of the case would reveal the true character of the affection.

The pathology of membranous croup is not yet a matter of precision. Reliable pathological researches have tended to establish that the submucous tissue along the air-passages is the true source of the plastic lymph, and that it passes by transudation through the mucous membrane and becomes concrete upon its outer surface. The natural product of this tissue under inflammation would be coagulable lymph, and Dr. Williams states that in examination of those who die "early in the disease, the submucous tissue between the rings of the trachea and in the lower parts of the larynx is much swelled." After the lymph has reached the mucous surface and becomes inspissated, "it is probably prevented from becoming organized, both by the motion of the trachea and by the mucous secretion with which it is intermixed." When the organization of the lymph is thus interrupted, there seems to be an effort of nature to effect its removal by a suppurative process.

Pathological observations of patients dying late in the disease, show the false membrane detached in irregular patches from the membrane of the trachea by the intervention of muco-purulent matter. Further observations tend to establish the fact that the fibrinous deposit invariably commences at the superior parts of the air-passages and extends downwards along the trachea, sometimes as far as the minute bronchial ramifications themselves.

A very large proportion of cases of membranous croup proves fatal. Out of 22 cases under charge of Dr. Ware, 19 died, and his high reputation as a physician warrants the conclusion that his practice was as judicious and the results as favorable as we ordinarily find in the treatment of this malady. This would show an average mortality of about 86 per ct. of all cases of membranous croup.

To analyze the treatment of the above case correctly, and give each remedy its appropriate credit of curative influence, is the most difficult part of my task. We are too apt to conclude at once, when a patient recovers after taking a remedy, that his recovery is because of the remedy. *Post hoc ergo propter hoc*, is an argument too inviting to be readily rejected by the physician. With my present views of croup I should adhere, under similar circumstances, to the prominent points of treatment developed in the case related.

I have not supposed that the nitrate of silver exercised any beneficial influence in the case—first, because the solution was too weak to be effectual; second, because the sponge was not passed sufficiently far to come in contact with the parts about the rima glottidis, where its application was most required; third, because no mitigation of symptoms seemed, in a reasonable time, to follow its use. I am not, however, disposed to undervalue the remedy. I believe it a most important addition to our means of treating croup. The recent report of the New York Academy of Medicine shows that the introduction of the sponge probang through the rima glottidis, even in the hands of those experienced in its use, is not a matter of extreme facility. Still, its practicability is acknowledged, and in the early stages of croup, before the false membrane has extended far into the trachea, it brings the remedy in direct contact with the affected part. It is possible, too, that the strong solution might be beneficial without entering the glottis, by lessening the irritability of the fauces and larynx, and thus serving to control the tendency to spasm. Its astringent effects upon the engorged mucous membrane may also be beneficial, and may be propagated by continuity farther than its immediate superficies of contact.

To the effects of calomel and hyoscyamus, I attach great importance in the remedial conduct of the case. At the commencement of the treatment the calomel was given in grain doses every two hours, with a view to produce purgation. After this it was diminished to half-grain doses, and these not oftener than every fourth hour, and administered only with a view to its alterative and antiplastic properties. Copious purgatives were avoided throughout the whole progress of the disease. The effects of the hyoscyamus were sought for to avert spasm of the glottis, and produce general quietude. That it answered these purposes I am firmly persuaded. Its effects were endeavored to be perpetuated, and the intervals between the doses were fixed with a view to keeping the patient, continually, somewhat under its influence. Eighteen grains of extract of hyoscyamus were administered during the first six days of treatment, after which period it was no longer given. It may be well to observe at this point, that from the resistance of the child a portion of his medicines was generally lost. The sulphate of zinc was a valuable means of expelling the tenacious mucus from the fauces, and may have facilitated the detachment of the membrane in the latter stages of the disease. At all events, emesis by this agent was not attended by any prostrating effects, and I should again employ it in a similar case.

With regard to the application of the water, I can only say that its use was with me entirely empirical. I do not know that I can offer any satisfactory explanation of its rationale, though I am convinced that its use was beneficial to my patient. It may be that the cold of its first contact may have lowered the local inflammation by abstracting temperature—then as the napkin grew warmer, and vaporization of the water began, the oil silk prevented its escape, so that it became heated from the warmth of the body, and produced the soothing, sedative influence of a tepid steam bath. And again, some of the best writers upon the subject of croup consider the inhalation of aqueous vapor an important succedaneum in its treatment. Almost all the vapor escaping from the cloths used, as I have indicated, would be over the upper and front part of the bandage, so that a portion of it must continually enter the lungs of the patient. Upon these considerations alone I would strongly recommend the use of the wet bandage.

The administration of the carbonate of soda in the latter stages of the disease, was based upon a supposition long since abandoned, that it favors the solution and breaking down of the fibrinous deposit.

To express an opinion of the treatment in the above case, in the aggregate, I should decide that it owed its favorable results—if they were due at all to the treatment—more to its conservative character than to any individual remedy or feature. No depressing remedies were long urged upon the patient—the continued influence of nauseants was avoided, and to this circumstance I attribute the fact that he took food willingly during most of the time that he was under treatment.

If Dr. Ware's opinion be correct, that the deposit of lymph in croup begins with the earliest noticeable symptoms of the disease, and it be true that when once effused it can only be removed by a process of suppuration, the inference would clearly follow that "perturbating and exhausting remedies" are not only at best of doubtful efficacy, but that they may prove deleterious by wasting the powers of life that are necessary to the successful issue of the suppurative process.

I fully concur with Dr. Ware in this opinion, and also that the rule of treatment in croup laid down in our authorities upon this subject, and which looks only to the lancet, purgatives, emetics, and nauseants, as curative agents, should be carefully reconsidered. It remains yet to be seen how much advantage Dr. Green's discoveries will afford us in the treatment of this disease. I think they merit the careful, honest attention of the whole profession.

I do not consider it necessary to occupy the time and attention of the Club by any remarks upon the treatment of other varieties of croup. Instances of mortality are uncommon in these forms of disease, and generally an emetic, a cathartic, or an anodyne, and the warm bath, constitute the only requisite treatment. It is probable that inflammatory croup would bear depletion with more safety if not benefit to the patient, than the membranous form

In the division of the disease adopted above, I have very nearly followed Dr. Ware, and have repeatedly drawn from his valuable essay upon croup. I had treated the case related in the previous pages upon the basis of a conclusion similar to his own before I had seen or been informed of his contributions upon this subject, and when I met with his paper his views of the disease seemed so logical and his language so perspicuous that it seemed to me very difficult to diverge widely from either and still adhere to truth.

In the treatment of this case I was assisted by three other physicians of the city, two of whom are members of this Association. It is due to them to express before the Club my thanks for the aid they courteously extended me.

Dr. Richardson doubted the possibility of a ringing cough when the vocal chords were covered with false membrane. It might be brassy.

Dr. Bemiss had seen 20 cases, of these only 2 recovered; and one of these got well, after several weeks of exhaustion, without treatment.

Dr. Wible, at Mt. Washington, in October, 1846, lost 7 cases in three weeks. They were all fatal under the antiphlogistic, calomel, and antimony treatment. One active and slender child threw up membrane two and half inches in length. He made no autopsies. The patients died in ten or eleven days from the commencement of the attack. Previously to the year in which these cases occurred, he had practiced nine years in the same neighborhood without seeing a case. He recollected another case, occurring at the same time, in the hands of another practitioner. It was also fatal.

Dr. Goldsmith had had but few cases in his own practice. In one case, during emesis, the false membrane was ejected. He related an instance in the practice of Dr. Godman, in which that physician had the good fortune to save his patient from immediate strangulation by placing his mouth to that of the child and sucking the membrane, which occluded the windpipe, into his own mouth.

Dr. Richardson had seen 1 case in his own practice—3 others in that of other physicians. All fatal. The 3 cases all occurred in three weeks—2 cases in robust children. He has seen 1 case of death from spasmodic croup. The patient died at the end of the sixth day. He found the pneumogastric nerve passing through two inflamed bronchial glands, and these glands in the course of the nerve in the neck were also inflamed.

Dr. Rogers could call to mind 9 cases, and all terminated fatally but 2; 1 recovered under the conservative treatment; all the others were treated antiphlogistically. One of the cases followed scarlatina. The children were all feeble. In all he noticed the false membrane upon the fauces.

Dr. Gross has practiced pretty extensively among children in Louisville for fifteen years. Recollects but 3 cases occurring here. One was fatal. Another, a child of Dr. Miller's, ejected false membrane and recovered. The treatment was alum emetics. The caustic was used, and the child well sustained. He saw several cases in Pennsylvania. In one case he assisted in a post-mortem, and the membrane extended into the bronchii. In Cincinnati he performed tracheotomy. The child died.

Dr. Knight had seen a number of cases, and some scarlatina cases followed by it. He saw only 1 case in which the membrane was expelled.

Dr. Ewing said when he lost a case of croup he generally considered it membranous. He thinks the most common cause of spasmodic croup is improper diet. He had frequently relieved these cases by freeing the stomach from irritating and indigestible substances.

Dr. Wible concurred with Dr. Ewing, and mentioned 2 cases corroborative of his opinion.

Dr. Bartlett had seen 2 cases; 1 followed scarlatina, and died without treatment. The other he saw seven days after the disease came on. The membrane was visible upon the fauces, and all the symptoms of true croup were present. Some membrane, he was told, had been expectorated. He gave alum emetics at the commencement, and afterwards sulphate of copper. He had swabbed out the larynx twice a day with a sponge saturated with a solution of nitrate of silver, at first 20 and afterwards 41 grains to the ounce. Patient recovered.

Drs. Richardson and Bemiss thought the English did not recognize well the difference between true and false croup.

Dr. Gross asked if the symptoms of croup might not be simulated by inflammation of the pharynx or œsophagus? False membranes form in this situation. He has a specimen of false membrane found upon these parts in a man of intemperate habits, who died with difficulty of respiration. This might have been the case in the patient of Dr. Godman.

Dr. Goldsmith was summoned to a negro child with symptoms of croup. The croupy cough and difficulty of breathing came on suddenly. Dr. G. stated his opinion that there was a foreign body in the air-passages, and advised an operation. The family refused. The child died, and upon examination a piece of bread was found in one of the bronchial tubes. It had been swallowed about half an hour previously.

On motion of Dr. Rogers, Dr. Bartlett was requested to read a paper on delirium tremens at the next meeting.

The amendment to the resolution proposed by Dr. Palmer, at the last meeting, was taken up and adopted.

On motion of Dr. Gross, it was resolved that none of the proceedings shall be published till they have been submitted to the Club at a regular meeting.

Dr. Wible's invitation to the Club to meet on the next evening at his office, was accepted, and the Club adjourned.

